# Physical activity programming and counseling preferences among cancer survivors: a systematic review

**DOI:** 10.1186/s12966-018-0680-6

**Published:** 2018-06-07

**Authors:** Jaime N. Wong, Edward McAuley, Linda Trinh

**Affiliations:** 1 Department of Kinesiology and Community Health, University of Illinois at Urbana-Champaign, Urbana, IL USA; 2 Faculty of Kinesiology and Physical Education, University of Toronto, 55 Harbord Street, Toronto, ON M5S 2W6 Canada

**Keywords:** Physical activity, Preferences, Cancer, Health behavior, Systematic review

## Abstract

**Background:**

Physical activity (PA) participation and adherence among cancer survivors is low, despite research indicating numerous physical, psychological and emotional health benefits of exercise. Tailoring exercise programs specific to the PA preferences in cancer survivors has merit for increasing PA participation and adherence to accrue these benefits. This systematic review identifies and differentiates PA programming and counseling preferences of adult cancer survivors across various cancer survivor groups.

**Methods:**

PubMed, SPORTDiscus, Scopus, PsycINFO, EMBASE, Web of Science and CINAHL were electronically searched (inception to Oct 2017) and articles were identified using PRISMA guidelines. Two reviewers independently assessed identified articles to determine eligibility and then individually performed a quality assessment on all final studies. Extracted and analyzed data included participant characteristics, interest in exercise counseling and programming, as well as specific exercise and counseling preferences (e.g. location, timing, intensity).

**Results:**

Forty-one articles were included in this systematic review. Most studies assessed mixed cancer survivor groups or breast cancer survivors. Most cancer survivors felt able and interested in participating in a PA program, though starting a PA program after or before treatment was preferred. Walking was the strongest PA modality preference, and most cancer survivors preferred moderate intensity PA. Cancer survivors also indicated preferences for home-based PA that could take place in the morning. Slight preferences were found towards physical activity counseling delivered by a fitness expert from a cancer center. Both quantitative and qualitative studies were found to be of moderate to high quality based on the Appraisal Tool for Cross-Sectional Studies (AXIS) and the Consolidated Criteria for Reporting Qualitative Research (COREQ), respectively.

**Conclusion:**

Cancer survivors have an interest in participating in PA programs with walking as the primary modality. Additionally, morning-based PA programs that can be tapered to home-based programs are desirable. However, there was wide variation in other PA preference variables, suggesting multiple program options would be beneficial. Many cancer survivors felt interested and able to participate in PA, and therefore designing PA programs that are tailored to cancer survivors is integral for optimizing recruitment and adherence, as well as enhancing health outcomes in cancer survivors.

**Electronic supplementary material:**

The online version of this article (10.1186/s12966-018-0680-6) contains supplementary material, which is available to authorized users.

## Background

Worldwide, one in three men and one in four women are expected to develop cancer in their lifetime [1]. Furthermore, between 2005 and 2015, the global incidence of cancer increased 33% and the incidence of cancer is expected to continue to rise. In spite of rising incidence rates, cancer mortality was found to decrease in many countries [1]. The rising rates of cancer survivors emphasize the need for supportive care programs. Physical activity (PA) has gained attention as a promising method of enhancing psychological, physical and emotional quality of life parameters for cancer survivors [2–8]. Evidence suggests that PA has numerous benefits for cancer survivors including positive effects on psychological, physical, and functional health [3, 9–16]. Moreover, physical activity has been found to reduce all cause and cancer-specific mortality among breast, prostate and colorectal cancer survivors [17–21]. However, cancer treatments may present additional challenges in adhering to a PA regimen and consequently, participation in PA has been shown to significantly decrease during treatment [22, 23].

International PA guidelines recommend that cancer survivors obtain 150 min of moderate aerobic PA per week, in addition to doing resistance training 2–3 times per week [24–26]. Despite the established benefits of PA, the majority of cancer survivors do not meet PA guidelines, and participation and adherence among cancer survivors is low [27–29]. During primary treatment, only 10% of cancer survivors are active, with 20–30% being active post-treatment [24]. Moreover, cancer survivors engage in significantly less PA compared to individuals with no previous cancer diagnosis [30]. To better understand determinants of PA, studies have investigated motivators and barriers to PA, finding that social cognitive variables (e.g. attitudes, barriers) correlate strongly to adherence [31–35]. It then follows that focusing on these variables when constructing a PA intervention can assist in raising adherence levels: tailoring a PA program to survivors’ preferences can help shape attitude and perceptions of PA [36].

Tailoring a PA program to the preferences of cancer survivors may have beneficial outcomes such as long-term PA maintenance [37, 38]. Given the numerous demographic, medical, and clinical differences across cancer survivor groups, PA type and intensity can be influenced by stage of cancer and type of treatment, as well as activity levels pre-diagnosis and thus, PA decisions should be personalized [24]. Previous studies have reviewed the PA preferences of cancer survivors: Syzmlek-Gay [39] performed a literature review assessing the PA preferences of cancer survivors, while Albrecht & Taylor [40] systematically reviewed PA outcomes in patients with advanced-stage, and included a review of PA interest and preference in this population. Cumulatively, these studies have suggested that most cancer survivors feel interested in either participating in a PA program and/or receiving information about a PA program. Although the preferences on the specifics of a program can vary, there may be commonalities as well. However, a systematic review that synthesizes all quantitative and qualitative PA preferences literature has yet to be conducted.

The purpose of this systematic review was to: a) summarize the current evidence on the interest and preferences of PA programming and/or counseling among adult cancer survivors; and b) identify and differentiate PA programming and counseling preferences across cancer survivor groups. The findings from this review can help to inform the design of future PA interventions that may optimize recruitment and adherence, as well as enhance health outcomes in cancer survivors.

## Methods

### Design

The reporting of this systematic review protocol adhered to the Preferred Reporting Items for Systematic Reviews and Meta-Analyses Protocol (PRISMA-P) checklist [41]. In addition, guidelines from the Cochrane Handbook for Systematic Reviews of Interventions and the Guidance on the Conduct of Narrative Synthesis in Systematic Reviews were used when applicable [42, 43].

### Search strategy

A search strategy was developed using an iterative process based on recommendations from a university research librarian. The databases PubMed, SPORTDiscus, Scopus, PsycINFO, EMBASE, Web of Science and CINAHL were electronically searched using keywords related to the PA preferences of adult cancer survivors as well as using Medical Subject Heading (MeSH) terms specifying participants (e.g. neoplasm, cancer survivor, cancer patient,), intervention (e.g. exercise, physical activity) and specific information sought (e.g. exercise preference, counsel, patient preference, exercise preference, health behavior counseling). PA can be defined as bodily movement of skeletal muscles that results in energy expenditure, whereas exercise is planned, structured and repetitive bodily movement with the goal of maintaining or improving fitness [44]. Both terms were included in the search string used, as in the MeSH system, ‘exercise’ encompasses ‘physical activity’. Reference lists of included articles were hand-searched for additional studies. Additional studies meeting inclusion criteria were located by assessing the reference lists of relevant reviews. Details of the search strategy are illustrated in the Appendix. Search results were exported, organized and de-duplicated within Mendeley (Elsevier, USA). A search log was maintained to record the initial search strategy and subsequent modifications, databases searched, and details on the identified studies.

### Inclusion criteria

Studies were included if they were published in English in peer-reviewed scientific journals from inception to October 2017. The types of studies, participants, and outcomes that were considered for inclusion are described below.

#### Types of studies

Studies of interest included those that examined PA programing and counseling preferences in cancer survivors. Empirical research studies using quantitative methods, intervention, or observational design were included, as well as studies using qualitative methods.

#### Types of participants

Studies were included if participants were adult cancer survivors, aged 18 years and older who were diagnosed with any cancer type and were at any point along the cancer care continuum at the time the study took place (i.e., diagnosis, treatment, post-treatment).

#### Types of outcome measures

Included studies examined at least one of the following primary outcomes: (1) PA programming preferences or (2) PA counseling preferences (i.e., studies that assessed the counseling or information delivery preferences of participants).

### Identification and selection of studies

After all potentially relevant studies were identified from each database, duplicates were removed and the titles and abstracts of the remaining studies were screened. Studies with titles and abstracts irrelevant to the research question were excluded first. If a study’s title suggested that it may contain relevant data, the abstract was assessed. Studies whose abstracts did not indicate relevance to this review were excluded. If the abstract indicated that the study collected information on the PA preferences of adult cancer survivors, the full-text article of the study was read to determine eligibility. The suitability of these remaining full-text articles were evaluated, with unrelated or non-applicable studies excluded following inclusion and exclusion criteria. Two independent reviewers (JW and LT) evaluated the identified articles to determine whether they would be included in the review. Disagreement between the two reviewers was resolved by discussion or consultation with a third reviewer when necessary.

### Data items extracted

Data was collected on participant characteristics from quantitative and qualitative studies (Table 1). Quantitative data relative to participant interest in PA programming, as well as preferences of when participants would prefer to start a program, general type of PA preferred, who counseling should be given by, how to receive counseling, intensity of preferred PA, PA companion preferences, PA programming location, time of day, and supervised versus unsupervised PA preferences are displayed in Table 2. The descriptive major themes of qualitative data were identified and synthesized in the results.

**Table 1:** Demographic, Clinical and Behavioral Characteristics of the Quantitative and Qualitative Studies

Study	Study Design	Mean Age	Mean BMI	Female %	White	Marital or Partner Status	% Stage I or II	Mean Months Since Diagnosis	Meeting or Above PA Guidelines	Geographic Location or Country	Highest Education Completed	Response Rate	Timing of Study	
Arthur et al., (2016) [75]	Quantitative	69.0	--	57.0%	84.0%	--	93.0%	--	--	Southeastern United States	Completed university/college or higher (34%)	71.8%	Newly diagnosed	
Bélanger et al., (2012) [68]	Quantitative	38.2	26.5	70.0%	87.4%	Married (62.0%)	--	73.6	51.0%^a^	Alberta, Canada	Completed university/college or higher (61.9%)	38.0%	Treatment/post-treatment	
Blaney et al., (2013) [78]	Quantitative	61.0 (Median)	29.0	76.0%	--	Married (70.9%)	40.2%	--	--	Northern Ireland	--	52.3%	Treatment/post-treatment	
Craike et al. (2017) [84]	Qualitative	62.0	--	54.0%	--	Living with a partner/spouse or partner/spouse and children (92.0%)	--	--	--	Victoria, Australia	Completed university/college or higher (42%)	--	Post-Treatment	
Culos-Reed et al., (2017) [73]	Quantitative	50.6	--	46.7%		Married/common law (86.7%)	0.0%	--	20.0% (T1 - pre treatment); 22.0% (2 months from T1)^a^	Alberta, Canada	Completed university/college or higher (73.3%)	45.7%	Postsurgery, before treatment	
Farrokhzadi et al. (2016) [65]	Quantitative	58.3 (Median)	27.1	100.0%	--	Married/de facto (59.0%)	--	--	53.0%^a^	Sydney, Australia	Completed university/college (40%)	24.0%	Diagnosed in the past two years	
Forbes et al., (2015) [69]	Quantitative	65.6	27.8	45.0%	97.0%	Married (80%)	79.0%	51.6	42.2%^a^	Nova Scotia, Canada	Postsecondary (51%)	38.0%	Treatment/post-treatment	
Gjerset et al., (2011) [76]	Quantitative	56.6		56.0%	--	--	--	42.9	--	Norway	Completed university/college or higher (16%)	67.0%	Post-Treatment	
Green et al., (2014) [70]	Quantitative	66.6	26.2	61.0%		Living with a partner (84.0%, prostate cancer); living with a partner (70.0%, breast cancer)	--	41.75	86.5%^a^	South-East Queensland and Northern New South Wales, Australia	Completed university/college (42%, prostate cancer); Completed university/college (39 breast cancer)	--	Treatment/post-treatment	
Harrington et al., (2013) [54]	Quantitative	72.0	29.2	0.0%	79.0%	Married or partnered (58.0%)	--	--	19.0%^a^	Arizona, United States	Greater than or equal to 12 years (52%)	--	Mid-treatment (receiving ADT)	
Jones et al., (2002) [36]	Quantitative	60.8	--	59.0%	--	Married/common law (79.1%)	77.5%	--	16.0%^b^	Alberta, Canada	Completed university/college or higher (46.7%)	53.0%	Treatment/post-treatment	
Jones et al., (2007) [80]	Quantitative	44.8	--	50.9%	--	Married/common law (76.5%)	22.6%	28	--	North Carolina, United States	Completed university/college (82.1%)	28.0%	Post-Treatment	
Kartolo et al., (2016) [48]	Quantitative	--	--	40.0%	62.0%	Married or living with a partner (59.3%)	--	--	--	Toronto, Canada	Completed university/college or higher (41.7%)	73.0%	Treatment/post-treatment	
Karvinen et al., (2006) [62]	Quantitative	64.5	--	100.0%	--	Married/common law (69.4%)	72.0%	52	--	Alberta, Canada	Completed university/college or higher (37.6%)	50.2%	No timing; cancer diagnosis	
Karvinen et al., (2007) [74]	Quantitative	--	--	25.7%	--	Married/common law (79.1%)	--	--	20.9%^b^	Alberta, Canada	Some post secondary or more (37.8%)	51.0%	No timing; cancer diagnosis	
Karvinen et al., (2011) [49]	Quantitative	57.1	31.8	100.0%	46.0%	Married (51.0%)	70.0%	14.9	21.0%^b^	North Carolina, United States	More than high school (56%)	88.0%	Treatment/post-treatment	
Karvinen et al., (2016) [50]	Quantitative	62.5	26.5	25.6%	71.4%	Married (61.9%)	--	--	16.7%^b^	North Carolina, United States	More than high school (38.1%)	81.1%	Before treatment	
Leach et al., (2015) [85]	Quantitative	66.4		53.0%	--	Married (78.5%)	24.3%	31.7	--	Alberta, Canada	--	71.7%	Treatment/post-treatment	
Lin et al., (2013) [82]	Quantitative	61.4	23.6	46.9%	--	Married (79.0%)	44.4%	26.14	--	Northern and Southern Taiwan	Completed university/college or higher (30.9%)	--	Post-Treatment	
Lowe et al., (2010) [51]	Quantitative	61.5	24.4	60.0%	--	Married/common law (42.0%)	--	--	--	Alberta, Canada	Completed grade 12 or higher (50%)	19.0%	Treatment/post-treatment	
Lowe et al., (2016) [47]	Quantitative	63.5	27.5	58.0%	--	Married/common law (74.0%)	--	--	--	Alberta, Canada	Completed grade 12 or higher (58%)	28.0%	Treatment	
McGowan et al., (2013) [71]	Quantitative	67.3	--	41.7%	94.3%	Married (74.0%)	24.2%	51	33.0%^a^	Alberta, Canada	Some post secondary or more (51%)	34.0%	Diagnosed between 2003-2007	
Midgley et al., (2017) [81]	Quantitative	66.0	--	26.0%	--	--	--	43.0 (Median)	--	North West England, United Kingdom	--	43.0%	Received treatment between 2010 and 2014	
Murnane et al., (2012) [52]	Quantitative	58.8	--	39.1%	--	--	--	--	--	Australia	--	77.0%	Before treatment/treatment	
Paxton et al., (2014) [58]	Quantitative	54.0	30.2	100.0%	0.0%	Married (49.1%)	78.8%	85.8	46.4%^a^	Online, United States (CA, FL, GA, IL, IN, LA, MD, MI, MS, NV, NJ, NY, NC, OH, SC)	Completed university/college or higher (52.2%)	--	Off treatment except hormone therapy	
Philip et al., (2014) [53]	Quantitative	68.7	25.9	63.4%	92.6%	Married/partnered (62.3%)	--	43.44	25.14%^a^	New York, United States	Completed university/college or higher (50%)	63.6%	Post-treatment	
Phillips et al., (2017) [66]	Quantitative	60.7	26.5	100.0%	97.1%	Married/partnered/significant other (78.9%)	--	139.2	53.4%^a^	United States (recruited nationwide)	Completed university/college or higher (71.7%)	20.4%	Post primary treatment	
Robertson et al., (2017) [86]	Qualitative	63.7		69.0%	71.0%	--	--	--	--	Texas, United States	Completed university/college or higher (63%)	--	Post Primary Treatment	
Rogers et al., (2007) [57]	Quantitative	--	--	100.0%	91.0%	--	74.0%	--	--	Illinois, United States	Equal to or greater than 16 years of education (24%)	88.5%	Treatment	
Rogers et al., (2008) [56]	Quantitative	64.4	27.5	100.0%	98.0%	--	50.0%	62.5	--	Midwest, United States	--	32.0%	Diagnosed between 1997-2002	
Rogers et al., (2009a) [79]	Quantitative	--	24.6	22.0%	96.0%	--	19.0%	--	--	Midwest, United States	Greater than or equal to 12 years (30%)	83.0%	No timing, just had to be diagnosed	
Rogers et al., (2009b) [59]	Quantitative	63.0	28.9	100.0%	96.0%	--	57.0%	39	19.0%^b^	Midwest, United States	--	86.0%	Diagnosed before 2005	
Spence et al., (2011) [55]	Qualitative	57.8	26.8	30.0%	--	--	--	--	--	Brisbane, Australia	--	--	Post-Treatment	
Stevinson et al., (2009) [64]	Quantitative	60.2	27.1	100.0%	--	Married/common law (73.5%)	45.4%	73.6	31.1%^b^	Alberta, Canada	Completed university/college or higher (42.3%)	51.4%	Diagnosed between 1985-2002	
Sturgeon et al. (2017) [67]	Quantitative	55.3	28.4	100.0%	71.6%	--	89.6%	--	35.4%^a^	Pennsylvannia, United States	--	69.6%	No timing; cancer diagnosis	
Tirado-Gomez et al., (2016) [60]	Quantitative	57.2	29.4	100.0%	--	Married/partnered (33.3%)	--	--	--	Puerto Rico	Completed university/college or higher (32%)	--	Post-Treatment	
Trinh et al., (2012) [72]	Quantitative	65.0	28.5	37.1%	91.0%	Married/common law (73.6%)	--	69	26.0%^a^	Alberta, Canada	Completed university/college or higher (40.4%)	42.5%	Diagnosed between 1996-2010	
Tyrrell et al., (2014) [63]	Quantitative	52.9	--	100.0%	93.0%	Married/common law (68.0%)	--	76.3	--	Nova Scotia, Canada	Completed university/college (28%)	62.0%	Diagnosed after 2001	
Vallance et al., (2006) [77]	Quantitative	61.1	26.3	48.4%	--	Married/common law (75.5%)	32.2%	62	--	Alberta, Canada	Completed university/college or higher (44.5%)	52.0%	Diagnosed between 1994-2001	
Vallance et al., (2013) [61]	Quantitative	62.4	26.6	100.0%	--	Married/common law (74.8%)	85.1%	76.4	34.7%^b^	Alberta, Canada	Completed university (49.8%)	30.0%	Post-treatment	
Whitehead & Lavelle (2009) [83]	Qualitative	66.5	--	100.0%	Mostly White European	--	--	--	--	Manchester and Derby, United Kingdom	--	--	Off treatment except hormone therapy	

Note: --information not assessed or reported

^a^Guidelines defined as 150 min moderate PA, 75 min of vigorous PA or a combination of the two

^b^Guidelines defined as 150 min moderate PA or at least 60 min of strenuous PA or a combination of the two

### Methodological quality assessment

Quality assessment was conducted using the Appraisal Tool for Cross-Sectional Studies (AXIS tool) for quantitative studies [45] and the Consolidated Criteria for Reporting Qualitative Research (COREQ) for qualitative studies [46]. Examples of items in the AXIS tool include assessing the appropriateness of study design for stated aims, sample size justification, the reliability of survey instruments, and evaluating whether the response rate raises concerns regarding non-response bias. The AXIS tool does not include a numerical scale that can be used to produce quality assessment score; instead, the tool aims assess the individual characteristics of a study cumulatively. The COREQ consists of 32 items, with higher scores indicating more thorough reporting. Qualitative studies are assessed through three domains: 1) research team and reflexivity, 2) study design, and 3) analysis and finding. Two reviewers (JW and LT) independently conducted the assessments. When there was divergence in scoring, reviewers discussed the item further until reaching a consensus; if necessary, a third reviewer was consulted.

## Results

**Fig. 1:**
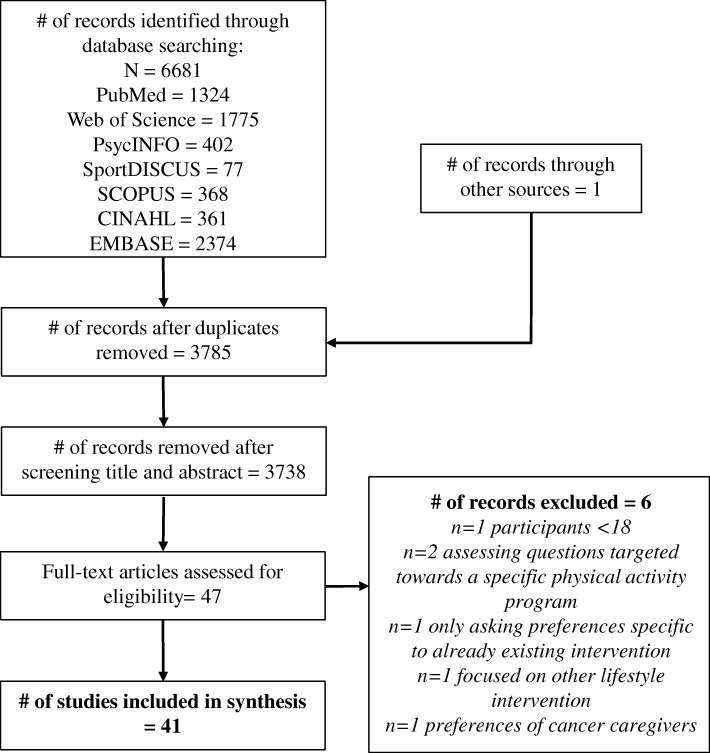
Study Flow Diagram

The electronic search yielded 6681 studies, with 1 study identified through other sources (i.e., reference lists). After de-duplicating records, 3785 articles remained. Further evaluation of titles and abstracts found that 3738 did not meet the inclusion criteria. Of these, 47 full-text articles were obtained for detailed eligibility assessment. Six were excluded for not meeting eligibility criteria. Finally, forty-one articles were included in this systematic review (Fig. 1).

### Study characteristics

Studies most commonly assessed mixed cancer survivor groups (*n* = 10) and breast cancer survivors (*n* = 10), followed by lung (*n* = 5), brain (*n* = 2), colorectal (*n* = 2), gynecologic (*n* = 2), head and neck (*n* = 2), endometrial (*n* = 1), bladder (*n* = 1), kidney (*n* = 1), non-Hodgkin’s lymphoma (*n* = 1), ovarian (*n* = 1), pancreatic (*n* = 1), multiple myeloma (*n* = 1), and prostate (*n* = 1). Of the studies assessing mixed cancer survivors, survivors were diagnosed with a wide range of cancer types including breast, prostate, colorectal, lymphoma, gynecological, hematological, sarcoma, lung, head and neck, non-Hodgkin lymphoma, leiomyosarcoma, genitourinary, gastrointestinal, melanoma, prostate, and thyroid cancer. Cancer survivors were assessed across the cancer continuum, though certain studies focused at specific time-points (i.e., newly diagnosed, before/after treatment, during treatment, cancer metastases, palliation) [47–55].

### Participant characteristics

The mean age of participants ranged from 38.2 to 72.0 years old, with an average gender distribution of 64.9% female and 35.1% male participants. One study consisted of only male participants – prostate cancer survivors [54], while thirteen studies consisted of solely female participants – gynecologic, ovarian, endometrial and breast cancer survivors [49, 56–67]. The mean body mass index (BMI) of participants ranged from 23.6 to 31.8 (mean = 27.3 kg/m^2^). Study sample sizes ranged from 10 to 1284, for a total of 10,530 participants across all studies. Between 16 and 88% participants were meeting PA guidelines (mean = 34.2%; median = 31.1%) (Table 2). Studies defined the guidelines in one of two ways: 1) 150 min of moderate physical activity or 75 min of vigorous physical activity per week [53, 54, 58, 65–73], or 2) 150 min of moderate-strenuous PA per week (i.e., an average of 60 min of strenuous activity or 150 min of moderate activity) [36, 49, 50, 59, 61, 64, 74]. The mean months since diagnosis ranged from 14.9 to 139.2. There was a large range of participants in different cancer stages (mean participants in Stage I or Stage II = 52.6%; median = 50%), excluding studies that specifically focused on palliative patients [51] and patients with brain metastases [47].

### Physical activity preferences outcomes

#### Interest in physical activity programming (*n* = 29)

Of the 41 studies, 29 assessed interest in PA programming. Quantitative results are presented in Table 2. In quantitative studies (*n* = 27), interest in PA was assessed in various ways, including: “[are you] interested in a program that would increase PA level?”, “[are you] interested in a PA program now?”, “would you have liked to receive information about participating in an exercise program?”, “any time before or after treatment, would you have been interested in taking part in an exercise program tailored to [cancer survivor group]?}” [47, 50, 51, 56, 58, 59, 61–65, 67–72, 74–82]. Participants across the majority of quantitative and qualitative studies expressed interest in participating in a PA program (*n* = 26) [50, 51, 53, 56, 58, 59, 61–65, 67–72, 74–79, 82–84]. In twenty-four quantitative studies, most participants indicated that they were or may be interested in participating in a PA program [50, 51, 53, 56, 58, 59, 61–65, 67–72, 74–79, 82].

**Table 2:** Study Descriptions and Physical Activity Preference Outcomes

Study	Design	Cancer Type	Interested in Physical Activity Program?	Program Start**	Physical Activity Most Interested In**	Counseling/Information Delivery**	Intensity	Physical Activity Companion**	Physical Activity Location**	Time of Day	Supervised/Instructed vs. Unsupervised/Self-paced Physical Activity	Ability to do Physical Activity Program
Arthur et al., (2016) [75]	Cross-sectional researcher-administered telephone survey (*n*=50)	Pancreatic	Yes (69.0%) (exercise and diet)	--	--	--	--	--	--	--	Unsupervised (50.0%)	--
Bélanger et al., (2012) [68]	Cross-sectional self-adminisered survey (*n*=588)	Mixed	Yes (48.0%)	3-6 months after treatment (33.5%)	Walking (51.3%, 40.1% in summer and winter respectively)	Fitness expert from cancer center (49.6%)	--	No preference (34.8%)	Community fitness center (53.5%)	--	--	Yes (62.2%)
Blaney et al., (2013) [78]	Cross-sectional self-administered survey (*n*=456)	Mixed	Yes (50.2%)	One year or more after completion (33.9%)	Walking (76.7%)	Specialist nurse (35.0%)	Moderate (60.2%)	Other cancer survivors (40.8%)	No preference (38.8%)	Morning (36.6%)	--	Yes (52.5%)
Culos-Reed et al., (2017) [73]	Cross-sectional survey (*n*=16)	Brain (High Grade Glioma)	--	During treatment (56.3%)	Walking (56.3%)	Exercise specialist from cancer center (62.5%)	Moderate (86.7%)	Alone (43.8%)	Home (42.9%)	Morning (53.3%)	Unsupervised/self-paced (71.4%)	--
Farrokhzadi et al. (2016) [65]	Cross-sectional self-administered survey (*n*=101)	Gynecologic	Yes (36%)	3-6 months after treatment (26%), During treatment (23%)	Walking (71%), swimming (31%)	--	--	Family/friends (37%), alone (26%)	--	--	--	--
Forbes et al., (2015) [69]	Cross-sectional self-administered survey (*n*=741)	Mixed	Yes (32.0%)	3-6 months after treatment (34.0%)	Walking*∞	Fitness expert from cancer center (51.0%)	Moderate (65.0%)	Friends (53.0%)	Outside around neighborhood (67.0%)	Morning (55.0%)	Unsupervised/self-paced (53.0%)	Yes (47.0%)
Gjerset et al., (2011) [76]	Cross-sectional self-administered survey (*n*=1284)	Mixed	Yes (67.0%)	Immediately after treatment (46.0%)	Walking (33.0%)	Exercise specialist from cancer centre (53.0%)	Moderate (77.0%)	No preference (32.0%)	Community fitness center (35.0%)	Morning (34.0%)	Supervised (64.0%)	Yes (74.0%)
Green et al., (2014) [70]	Cross-sectional self-administered survey (*n*=237)	Mixed	Yes (57.0%, prostate cancer); Yes (61.0%, breast cancer)	At diagnosis or soon after (prostate, 58.0%; breast, 49.0%)	--	--	--	--	--	--	--	--
Harrington et al., (2013) [54]	Cross-sectional researcher-administered interview (*n*=135)	Prostate	--	--	Walking (64.0%)	--	Moderate (56.0%, 64.0% for aerobic exercise and muscle strengthening respectively)	Alone (37.0%, 43.0% for aerobic exercise and muscle strengthening respectively)	Home (52.0%, 55.0% for aerobic exercise and muscle strengthening respectively)	--	Unsupervised (57.0%, 59.0%, for aerobic exercise and muscle strengthening respectively)	Yes (79.0%)
Jones et al., (2002) [36]	Cross-sectional self-administered survey (*n*=307)	Mixed	--	Before treatment (31.8%)	Walking (80.6%)	Exercise specialist from cancer centre (76.8%)	Moderate (56.1%)	Alone (43.6%)	At home (39.8%)	Morning (47.5%)	Unsupervised (57.4%)	--
Jones et al., (2007) [80]	Cross-sectional self-administered survey (*n*=106)	Brain	During Treatment: Yes (29.2%); After treatment: Yes (55.7%)	--	During Treatment: Walking (51.0%); After Treatment: Walking (53.0%)	--	--	During Treatment: No preference (26.4%); After Treatment: Spouse/family (40.6%)	During Treatment: At home (25.5%); After Treatment: At home (43.4%)	--	--	During Treatment: Yes (30.2%); After Treatment: Yes (65.1%)
Kartolo et al., (2016) [48]	Cross-sectional administrator unreported (*n*=60)	Lung	--	--	Walking (80.0%)	--	--	Alone (49.0%)	Home (67.0%)	--	--	--
Karvinen et al., (2006) [62]	Cross-sectional self-administered survey (*n*=386)	Endometrial	Yes (41.5%)	3-6 months after treatment (39.3%)	Walking (68.6%)	Exercise specialist from cancer centre (40.9%)	Moderate (61.1%)	No preference (32.7%)	At home (32.7%); No preference (32.7%)	Morning (51.3%)	Supervised (53.1%)	Yes (46.4%)
Karvinen et al., (2007) [74]	Cross-sectional self-administered survey (*n*=397)	Bladder	Yes (44.5%)	Immediately after treatment (39.1%)	Walking (81.1%)	Exercise specialist from cancer centre (40.1%)	Moderate (61.7%)	No preference (36.3%)	At home (53.7%)	Morning (36.6%)	Unsupervised (70.6%)	Yes (47.0%)
Karvinen et al., (2011) [49]	Cross-sectional researcher-administered survey (*n*=91)	Breast	--	--	Walking (61.0%)	Exercise specialist from cancer centre (71.0%)	Moderate (47.0%)	Other cancer survivors (25.0%)	At home (37.0%)	--	Supervised (66.0%)	--
Karvinen et al., (2016) [50]	Cross-sectional researcher-administered survey (*n*=43)	Lung	Yes (58.1%)	During chemotherapy (51.3%)	--	Oncologist (27.0%)	Moderate (55.0%)	Family/friends (42.5%)	Home (56.8%)	--	--	Yes (58.1%)
Leach et al., (2015) [85]	Cross-sectional self-administered survey (*n*=66)	Lung	--	Before treatment (25.7%)	Walking (79.5%)	Exercise specialist from cancer centre (59.4%)	Moderate (62.2%)	In group of cancer survivors (28.6%)	Cancer exercise center (26.8%)	Morning (56.1%)	Supervised (62.2%)	--
Lin et al., (2013) [82]	Cross-sectional self-administered or researcher-administered survey (*n*=81)	Lung	Yes (70.4%)	3-6 months after treatment (23.5%)	Walking (88.9%)	No preference (49.4%)	--	Alone (44.4%)	Outdoors (54.3%)	Early morning (53.1%)	Unsupervised (64.2%)	Yes (69.1%)
Lowe et al., (2010) [51]	Cross-sectional researcher-administered survey (*n*=50)	Mixed	Yes (78.0%,)	--	Walking (64.0%)	--	--	Alone (54.0%)	Home (84.0%)	Morning (40.0%)	--	Yes (58.0%)
Lowe et al., (2016) [47]	Cross-sectional researcher-administered survey (*n*=27)	Mixed	Yes (19.0%)	--	Walking (45.0%)	--	--	Family/friends (29%); No preference (29%)	Home (58.0%)	Morning (52.0%)	--	Yes (39%)
McGowan et al., (2013) [71]	Cross-sectional self-administered survey (*n*=600)	Colorectal	Yes (47.0%)	3-6 months after treatment (28.0%)	Walking or hiking (49.0%, 37.0% in summer and winter respectively)	Fitness expert from cancer center (47.0%)	--	No preference (32.0%)	Home (56.0%)	--	--	Yes (50.0%)
Midgley et al. (2017) [81]	Cross-sectional self-administered survey (*n*=437)	Head and Neck	Yes (30%); Maybe (34%)	No preference (30%); Not sure (22%); 1 year or more after treatment (18%)	Walking (68%); Flexibiity exercises (35%)	--	Moderate (49%)	--	Home (55%); Outdoors (46%)	--	--	Yes (90% of participants with strong interest; 30% of participants with lesser interest)
Murnane et al., (2012) [52]	Cross-sectional self-administered survey (*n*=92)	Mixed	--	Before treatment (41.3%)	--	Exercise specialist/physiotherapist associated with cancer center (44.0%)	--	--	Home (52.9%)	--	--	--
Paxton et al., (2014) [58]	Cross-sectional self-administered survey (*n*=475)	Breast	Yes/Maybe (67.0%)	--	Walking (32.0-43.0%)	--	--	No preference (64.0%)	--	--	--	Yes/maybe (90.0%)
Philip et al., (2014) [53]	Cross-sectional telephone and mail survey (*n*=175)	Lung	Yes (52.0%)	Before treatment (60.0%)	Walking (42.0%)	Physician (80.0%)	Moderate (44.0%)	No preference (49.0%)	At a gym (29.0%)	--	--	Yes (74.0%)
Phillips et al. (2017) [66]	Cross-sectional self-administered survey (*n*=270)	Breast	--	--	Walking (69.5%); resistance training (68.1%)	Exercise specialist from cancer center (30.2%) (preference for remotely delivered counseling)	--	Combination of alone and group sessions (57.7%)	Outdoors (36.2%); home (25.1%), health club (24.4%)	--	Supervised by an exercise specialist initially, then unsupervised (26.3%), Supervised by an exercise specialist (25.5%)	--
Rogers et al., (2007) [57]	Cross-sectional researcher-administered (*n*=23)	Breast	--	--	Walking (100.0%)	--	Moderate (61.0%)	Alone (44.0%)	Home (48.0%)	Early evening or at night (39.0%)	Unsupervised (57.0%)	--
Rogers et al., (2008) [56]	Cross-sectional self-administered survey (*n*=192)	Breast	Yes (38.0%)	--	Walking (65.0%)	No preference (40.0%)	Moderate (64.0%)	Alone (41.0%)	Home (36.0%)	Morning (52.0%)	Unsupervised (49.0%)	Yes (64.0%)
Rogers et al., (2009a) [79]	Cross-sectional self-administered survey (*n*=90)	Head and Neck	Yes (33.0%)	--	Walking (47.0%, 44.0% in summer and winter respectively)	No preference (66.0%)	Moderate (50.0%)	Alone (50.0%)	Outdoors (47.0%)	Morning (47.0%)	Unsupervised (55.0%)	Yes (51.0%)
Rogers et al., (2009b) [59]	Cross-sectional self-administered survey (*n*=483)	Breast	Yes (33.0%)	--	Walking (55.0%, 41.0% in summer and winter respectively)	No preference (49.0%)	Moderate (65.0%)	Alone (41.0%)	Home (38.0%)	Morning (53.0%)	Unsupervised (47.0%)	Yes (55.0%)
Stevinson et al., (2009) [64]	Cross-sectional self-administered survey (*n*=359)	Ovarian	Yes (53.8%)	3-6 months after treatment (25.8%)	Walking (62.7%)	--	--	Friends/family (30.5%)	Home (48.9%)	Morning (48.9%)	--	Yes (64.6%)
Sturgeon et al. (2017) [67]	Cross-sectional researcher-administered survey (*n*=67)	Breast	Yes (76.2%)	--	--	Cancer exercise specialist (47.7%)	--	--	--	--	--	--
Tirado-Gomez et al., (2016) [60]	Cross-sectional self-administered survey (*n*=50)	Breast	--	--	--	--	--	Group setting (72.0%)	--	--	--	--
Trinh et al., (2012) [72]	Cross-sectional self-administered survey (*n*=703)	Kidney	Yes (43.8%)	3-6 months after treatment (36.5%)	Walking (69.4% summer, 48.2% winter)	Fitness expert from cancer center (55.7%)	Moderate (58.4%)	Spouse (39.2%)	Home (52.0%)	Morning (58.3%)	Unsupervised/self-paced (59.2%)	Yes (48.0%)
Tyrrell et al., (2014) [63]	Cross-sectional self-administered survey (*n*=239)	Gynecologic	Yes (37.0%)	3-6 months after treatment (68.0%)	Walking (95.0%)	--	Moderate (84.0%)	Alone (79.0%)	Home (81.0%)	Morning (79.0%)	Supervised (72.0%)	Yes (46.0%)
Vallance et al., (2006) [77]	Cross-sectional self-administered survey (*n*=431)	Non-Hodgkin's Lymphoma	Yes (55.4%)	3-6 months after treatment (38.2%)	Walking (55.0%)	--	Moderate (62.0%)	No preference (34.0%)	Home (42.6%)	Morning (41.3%)	Unsupervised (58.8%)	Yes (52.9%)
Vallance et al., (2013) [61]	Cross-sectional self-administered survey (*n*=524)	Breast	Yes (48.8%)	Immediately after treatment (41.1%)	Walking (51.1%)	Physical activity specialist (36.8%)	Moderate (65.8%)	Other women my age (33.5%)	Community fitness center (57.6%)	Morning (59.0%)	Supervised/instructed (58.7%)	Yes (50.8%)

Note: --information not assessed or reported

**some studies allowed participants to indicate more than one response

*∞ listed as first of top 3 in open-ended question

Craike et al. [84] and Whitehead et al. [83] qualitatively found participants to generally be interested in participating in a PA program, but noted that some participants were disinterested or hesitant for a number of reasons such as concerns about the format of the program (i.e., disinterest in the proposed group activities) and treatment status (i.e., being uninterested in participating in the proposed program during treatment). Although most studies found positive interest in PA program participation, three quantitative studies found higher percentages of participants to indicate disinterest in participating in a physical activity program [47, 80, 81]. Lowe et al. [47] found 65% of patients with brain metastases were not interested in a PA program now. Jones et al. [80] found that slightly more brain cancer patients were not interested in receiving information about participating in a PA program during treatment (37.7%) than those that were interested (29.2%). However, when asked the same question after treatment, most participants were interested (55.7%) [80]. Additionally, although Midgley et al. [81] found most head and neck cancer survivors indicated that they were or may be interested in participating in a PA program (64%), a higher percentage of head and neck cancer survivors were disinterested in a PA program (36%) than interested (30%). Forbes et al. [69] found similar numbers of mixed cancer survivors interested in doing a *PA program for cancer survivors* (i.e., 32% responded “yes” and 32% responded “no”), while more participants (47%) were interested in *a program that would increase PA levels* [69].

#### Physical activity program start (*n* = 24)

Twenty-two quantitative studies asked participants when they would prefer to start a PA program and two qualitative studies reported program start preferences [36, 50, 52, 53, 55, 61–65, 68–74, 76–78, 81, 82, 84, 85]. There was a large range of preferences for when to start a program. Most commonly, studies (*n* = 10) found participants to prefer starting a program 3–6 months after treatment [62–65, 68, 69, 71, 72, 77, 82]. Of the nine high quality studies assessing this variable, six found that starting a program 3–6 months following treatment was the most common preference [63, 64, 68, 69, 72, 77]. Forbes et al. [69] found the preference of starting 3–6 months after treatment to remain consistent across cancer survivor groups, with breast, prostate and colorectal cancer survivors all individually indicating a preference for starting 3–6 months after treatment.

Nonetheless, there were exceptions to this trend: three studies assessing mixed cancer survivors, bladder cancer survivors and breast cancer survivors found their participants to prefer staring a PA program immediately after treatment [61, 74, 76]. Four studies, two assessing lung cancer survivors and two assessing mixed cancer survivors found their participants to prefer starting a PA program before treatment [36, 52, 53, 85] and three studies found survivors to prefer starting a PA program during treatment or at diagnosis or soon after [50, 70, 73] One study assessing mixed cancer survivors found most participants to prefer starting a program one year or more after completion of treatment [78]. Lung cancer survivors generally expressed a preference for starting PA before treatment or 3–6 months after treatment [53, 82, 85].

Spence et al. [55] and Craike et al. [84] qualitatively found participants to prefer starting a PA program after treatment with varying preferred time frames between the end of treatment and the start of a program. In both studies, participants expressed concern about how treatment-related symptoms and functionality would impact participation in a program.

#### Physical activity modality preference (*n* = 34)

The majority of the studies (*n* = 34) assessed the types of PA in which cancer survivors were most interested [36, 47–49, 51, 53–59, 61–66, 68, 69, 71–74, 76–85]. Walking was overwhelmingly preferred across survivor groups in all quantitative studies (*n* = 31), with most participants in each study designating it as the PA in which they were most interested [36, 47–49, 51, 53, 54, 56–59, 61–64, 68, 69, 71, 72, 74, 76–80, 82, 85]. This remained consistent among all high quality studies assessing this variable [51, 53, 61, 63, 64, 68, 69, 72, 74, 77]. However, four quantitative studies indicated additional preferences towards a muscle strengthening program or resistance training [54, 58, 66, 80]. Interest in resistance exercise was also found qualitatively by Spence et al. [55] and Craike et al. [84].

#### Physical activity counseling and information delivery preference (*n* = 23)

Most quantitative studies (*n* = 22) assessed where participants would prefer to receive PA information or counseling [36, 49, 50, 52, 53, 56, 59, 61, 62, 66–69, 71–74, 76, 78, 79, 82, 85] while one qualitative study investigated PA information delivery preferences [84]. Studies assessed how participants preferred to receive information/counseling from a range of choices, including fitness experts (i.e. PA specialist, exercise physiologist) from a community or a cancer center, nurses, oncologists or other cancer survivors. The majority of studies (*n* = 15) reported that most cancer survivors indicated they would prefer to receive PA counseling or information from a fitness expert associated with a cancer center or a PA specialist [36, 49, 52, 61, 62, 66–69, 71–74, 76, 85]. When assessing breast, prostate and colorectal cancer survivors, Forbes et al. [69] found each individual cancer survivor group to most commonly prefer receiving PA information from a fitness expert from a cancer center. Moreover, five of the six high quality studies assessing breast, kidney, lung, colorectal, bladder and mixed cancer survivors information delivery preferences found receiving information/counseling from a fitness expert from a cancer center to be the highest preference [61, 68, 69, 72, 74].

The second most common preference for PA counseling or information delivery was from a health practitioner (i.e., specialist nurse, physician, or oncologist) [50, 53, 78]. These findings were reflected in a qualitative study performed by Craike et al. [84] who found multiple myeloma survivors to indicate that they would most trust healthcare clinicians (i.e., general practitioners, hematologists, oncologists) with knowledge of multiple myeloma to relay PA information.

#### Physical activity companion preferences and social support (*n* = 35)

Thirty-two quantitative studies assessed PA companion preferences, asking participants who they would most prefer to do PA with and three qualitative studies discussed group PA programs [36, 47–51, 53–66, 68, 69, 71–74, 76–80, 82, 83, 85, 86]. Eleven studies indicated cancer survivors to most prefer exercising alone [36, 48, 51, 54, 56, 57, 59, 63, 73, 79, 82]. Nine studies found that most of the cancer survivors they surveyed expressed no preference for PA companion [47, 53, 58, 62, 68, 71, 74, 76, 77]; within five of these, the second highest preference was to exercise alone [53, 62, 71, 74, 77]. Three studies revealed most cancer survivors to prefer exercising with other cancer survivors and one study found participants to prefer exercising with “other women my age” [49, 61, 78, 85]. Although exercising alone was the strongest individual PA companion preference, a comparable number of quantitative studies (*n* = 11) [47, 49, 50, 60, 61, 64, 65, 69, 72, 78, 85] found participants to prefer exercising with a partner in general (i.e., family, friends, other cancer survivors, etc.) and one study found participants to prefer a combination of alone and group sessions [66]. Among high quality studies, there was no consensus on a preference for a specific physical activity companion. Aside from the two high quality studies that found participants to prefer doing PA alone [51, 63], four studies reported no preference [53, 68, 74, 77], two studies reported a preference towards friends [64, 69], one study reported a preference for “other women my age” [61] and one study reported a preference for doing PA with a spouse [72].

Participants in qualitative studies reported that receiving social support through a group PA program would be a beneficial aspect of participating in a program. Qualitative studies [55, 83, 86] found some participants to indicate that enrolling in a program with others would assist them in maintaining motivation and adherence or would provide them an avenue through which to share like experiences with. Participants expressed interest in participating in a program with other cancer survivors [55, 83], as activities could then be tailored to the specific needs of the cancer population. However, in all qualitative studies, there were also participants who reported disinterest in group activities or PA social experiences [55, 83, 86].

There were differences in which quantitative studies categorized companion preferences and which categories were listed as options. For example, when asking participants “Who to do PA with?” Forbes et al. [69] and Trinh et al. [72] listed “alone”, “other cancer survivors”, “family”, “friends”, and “spouse” as options to select, with family, spouse and friends listed as three distinct selections [69, 72]. Other studies such listed “Family/friends” as a single response [47, 50, 65, 68] or “family member/spouse” as a single response [58].

#### Physical activity program location (*n* = 34)

Thirty-one quantitative studies and three qualitative studies assessed PA program location preferences, asking participants where they would most like to be active (e.g., home, neighborhood, fitness center) [36, 47–57, 59, 61–64, 66, 68, 69, 71–74, 76–85]. Most quantitative studies (*n* = 20) across cancer types found cancer survivors to prefer exercising at home [36, 47–51, 54, 56, 57, 59, 62–64, 71–74, 77, 80, 81]. Among the majority high quality studies, preference for doing PA at home remained stable [51, 63, 64, 72, 74, 77]. Craike et al. [84] qualitatively found around half of participants to prefer a home-based physical activity program as this would improve the amount of flexibility an individual would have in working around medical appointments and other commitments compared to a set program. Out of ten high quality studies, four found participants to more frequently express an alternate location preference. For example, Vallance et al. [61] found rural breast cancer survivors to prefer a community fitness center, while Philip et al. [53] found lung cancer survivors to prefer doing PA at a gym. Spence et al. [55] qualitatively found colorectal cancer survivors to prefer the gym as well, as this location allows for a wide variety of machine selection, regulated temperatures, and easy potential to regulate exercise intensity. In a qualitative study by Whitehead & Lavelle [83], participants emphasized the importance of having PA location options that accommodate participants, as cancer survivors can face location barriers that may influence their ability to participate. Aside from interest in home-based PA, Craike et al. [84] qualitatively found many participants to prefer PA at the hospital where they had been treated or medically focused location, as a program provided by a hospital could be viewed as a supplement to treatment.

#### Time of day for physical activity/exercise program (*n* = 20)

More than half of the total number of quantitative studies (n = 20) asked participants when they would most prefer to participate in a PA program [36, 47, 51, 56, 57, 59, 61–64, 69, 72–74, 76–79, 82, 85]. All high quality studies assessing this variable (*n* = 8) [51, 61, 63, 64, 69, 72, 74, 77] and nearly all studies (*n* = 19) [36, 47, 51, 56, 59, 61–64, 69, 72, 74, 76–79, 82, 85] found cancer survivors to prefer morning PA programs over other options such as the afternoon or evening program. In contrast, Rogers et al. [57] found 39% of breast cancer patients to prefer PA in the early evening or at night, while 35% indicated early morning and 26% indicated during the day.

#### Supervised vs. unsupervised physical activity (*n* = 22)

Twenty quantitative studies and two qualitative studies assessed whether participants preferred unsupervised/self-paced PA versus supervised/instructed PA [36, 49, 54–57, 59, 61–63, 66, 69, 72–77, 79, 82, 84, 85]. Most quantitative studies (*n* = 13) found participants to prefer unsupervised PA [36, 54, 56, 57, 59, 69, 72–75, 77, 79, 82], while less than half (*n* = 6) preferred supervised PA [49, 61–63, 76, 85]. Among high quality studies assessing this variable, only two studies found participants to prefer supervised PA [61, 63] while the remaining four found participants to prefer a unsupervised PA over supervised format [69, 72, 74, 77]. These results contrast to those found qualitatively by Spence et al. [55] where participants expressed a preference towards supervised PA as this format would assist in holding them accountable and keeping them motivated. Craike et al. [84] qualitatively found the supervision preferences of participants to be evenly split. However, participants relayed that participation in a supervised PA program at a hospital could allow for supervision from medical professionals and socialization with people undergoing similar experiences.

#### Ability to participate in a physical activity program (*n* = 24)

Twenty-four quantitative studies asked participants whether they felt able to participate in a PA program [47, 50, 51, 53, 54, 56, 58, 59, 61–64, 68, 69, 71, 72, 74, 76–82]. In twenty-two studies, most participants felt like they were able or may be able to participate in a PA program, with 78–95% of participants expressing interest [50, 51, 53, 54, 56, 58, 59, 61–64, 68, 69, 71, 72, 74, 76–79, 81, 82]. Conversely, two studies revealed the majority of participants to feel unable to participate in PA [47, 80]. Jones et al. [80] found that during treatment, only 30.2% of patients with brain metastases felt able to participate in PA and 17% felt like they may be able to participate in PA; most patients (32.1%) felt incapable. However, when assessing PA ability after treatment, their results more closely reflected the results other studies, with 65.1% feeling able to participate in PA, 18.9% feeling like they may be able to participate in PA and only 8.5% feeling incapable. Lowe et al. [47] found that the majority of patients in a mixed cancer sample of patients with brain tumors (58%) did not feel that they were able to participate in a PA program now.

#### Individualization or tailoring (*n* = 4)

Across all four of the qualitative studies assessed in this review, a theme of participant interest in PA program individualization or tailoring emerged [55, 83, 84, 86]. Spence et al. [55] found colorectal cancer survivors to express a preference towards having a choice of activity for a PA program, given that individuals find enjoyment in different activities. Whitehead & Lavelle [83] and Craike et al. [84] both found participants to find tailoring/individualization and flexibility in a program to be important because of the variation in PA ability and side effects of treatment. In assessing preferences for a PA mobile health app for cancer survivors, Robertson et al. [86] found participants to highly favor user-individualization, with tailoring around factors such as cancer-related information, personal health concerns, PA preferences and limitation, physical limitations, and age.

### Quality assessment

The quality of the studies assessed in this review appeared to range from moderate to strong, as all 37 quantitative studies met majority of the AXIS tool criteria [45], meeting 16 to 20 out of 20 possible items (mean = 18.7 ± 1.1) (see Additional file 1). Of these 37 studies, 10 met 20 out of 20 criteria [51, 53, 61, 63, 64, 68, 69, 72, 74, 77]. Of the studies that missed items, most did not take measures to address and categorize (*n* = 15, 40.5%) or describe non-responders (*n* = 25, 67.6%). Reviewers’ evaluations matched 75–100% in each study. All qualitative studies were of moderate to high quality, meeting 25 to 28 COREQ criteria [46] out 32 possible items (mean = 26.3 ± 1.5) (see Additional file 1) [55, 83, 84, 86]. Generally, studies missed items such as not reporting whether field notes were taken, whether participants were given the transcripts of the interview for comment and/or correction, and whether participants provided feedback on the study’s findings. Reviewers’ evaluations matched 81–88% in each study.

## Discussion

This systematic review provides a summary of the current evidence on PA programming and counseling preferences in cancer survivors to facilitate the adoption and maintenance of PA. Knowledge of unique interests and preferences is warranted for successful behavior change as interventions are more effective if they are tailored to the patient population.

Although studies indicated that cancer survivors have strong commonalities in preferences, results also suggested that there are large variations in PA programming and counseling preferences. Overall, the strongest preference centered on PA modality, intensity and preferred time of day. Nearly all studies indicated a specific preference towards walking as a preferred mode of PA [36, 47–49, 51, 53, 54, 56–59, 61–66, 68, 69, 71–74, 76–82, 85], suggesting that programs for cancer survivors should be designed around walking to increase interest in participation. Additionally, most studies found that cancer survivors may have a preference for PA programs held in the morning [36, 47, 51, 56, 59, 61–64, 69, 72–74, 76–79, 82, 85], as well as a preference for moderate-intensity PA programs [36, 49, 50, 53, 54, 56, 57, 59, 61–63, 69, 72–74, 76–79, 81, 85]. General preferences towards home-based and unsupervised programs were also reported. A meta-analysis by Buffart et al. (2017) examining the effects of PA on quality of life and physical function found supervised PA to have twice the effect size of unsupervised PA and suggests that this can potentially be attributed to greater guidance from a trainer, access to equipment, and adherence to protocol [87]. Developing home-based interventions or interventions that eventually transition to home-based PA may be beneficial for cancer survivors, in light of their preferences. However, it is important to note the distinction between unsupervised and home-based programs. Home-based programs can include motivational support, guidance and counseling [88, 89] through m-health, e-health and telephone-based interventions. Similarly, a program can be center-based and unsupervised. Results from this review suggest cancer survivors prefer moderate-intensity PA. However, programs should consider starting with moderate intensity PA and gradually increasing to high intensity PA, as a recent systematic review found that high-intensity PA can alone, or in combination with resistance training, lead to significant improvements in cardiovascular fitness and strength in cancer survivors with low risk of adverse events and reduced time commitment [90].

Significant variations were found in PA preferences, suggesting that multiple program options to accommodate these findings would be ideal. For example, there was no consensus on preferred physical activity companion. Generally, studies were divided, with around a third of quantitative studies finding cancer survivors to predominately prefer exercising alone, to have no preference, or to exercise with a companion. Moreover, amongst the studies that reported a distinct preferred companion (i.e., family, friends, other cancer survivors, groups), there was no specific companion option that was distinctly commonly preferred across studies [47, 49, 50, 60, 61, 64, 65, 72, 78, 85] This is especially observable in a study performed by Forbes et al. [69], evaluating the preferences of breast, prostate and colorectal cancer survivors. The preferences of each cancer group were compared and significant differences were found between groups when assessing PA companion preferences, namely, engaging in PA with other cancer survivors or with a friend.

Variation in preferences was also reported for PA program start. Most cancer survivors indicated a preference for starting a PA program when they were not currently going through treatment [36, 52, 53, 61–65, 68–72, 74, 76–78, 82, 85]. However, there was little consensus on the specific time period of 3–6 months after treatment, before treatment or immediately after treatment. Despite this, cancer survivors appeared to be aware that enrolling in a program would benefit them and that starting a program immediately post-treatment may assist with minimizing the risk of not starting [55]. It is important to acknowledge that although most cancer survivors indicated a preference for participating in a program while not undergoing treatment, this may be due to a lack of awareness of the benefits of PA during treatment, or lack of recommendation from health care providers to their patients in recommending PA during treatment [91].

Although most cancer survivors were not meeting PA guidelines, most studies found participants to be interested in participating in PA programs as well as feel able to participate, emphasizing the need for PA programs designed for cancer survivors. However, PA preferences, may be influenced by context and timing – for example, some studies asked participants interest in specific PA program formats [83] (i.e., interest in a group program with other cancer survivors) or about interest in a program during specific phases of the cancer continuum [84] (i.e., during treatment), which could affect preferences.

Few programming or counseling preferences differed by cancer type. Trends in PA preferences were difficult to determine due to a combination of reasons: 1) most studies were conducted with mixed cancer survivor groups, 2) only breast and lung cancer survivor groups had multiple studies assessing preferences, and 3) many studies did not ask participants the same questions, making it difficult to discern trends, especially within studies assessing breast cancer and lung cancer as these were the two most frequently studied cancer survivor groups. However, there were a few potential trends among breast cancer survivors and lung cancer survivors that warrant mention. Most lung cancer survivors [48, 53, 82, 85] indicated a preference towards starting a PA program before treatment or 3–6 months after treatment. Studies evaluating breast cancer survivors’ PA counseling preferences [56, 59] found them to either have no preference on who to receive counseling from or to prefer an exercise/PA specialist.

Although studies were generally of moderate to high quality, there was little standardization across studies in terms of the preferences assessed, the terminology chosen to assess preferences, preference options, and selection options (i.e., multi-select versus single-select). Lack of question standardization may have influenced participant responses when comparing studies. For example, when studies evaluated whether participants felt able to participate in a PA program, there was a range of terminology. While a few studies asked participants if they felt able to participate in a PA program specifically for cancer survivors/cancer survivor group, other studies asked whether participants they felt able to participate in a moderate-intensity exercise program or asked if participants felt capable of engaging in an exercise program. This choice in terminology may have been important, as assessing whether participants felt able to participate in an exercise program versus assessing whether participants felt able to participate in an exercise program specifically designed for their cancer survivor group, may influence how participants interpret the question.

Designing programs based on the preferences of cancer survivors may be important to increase interest and adherence to PA programs. However, caution should be taken when developing conclusions based on preferences research, as the PA preferences cancer survivors delineate may be partially dependent on their level of PA experience. For example, a cancer survivor who has limited PA experience may not know their preferences until they engage in PA and many of the studies assessed did not ask participants whether they were aware of the PA guidelines for cancer survivors. Future PA preferences research should consider assessing participants’ preferences before and after an intervention, or consider formative research to assess preferences for an intervention before designing the intervention to ensure it is tailored to this population.

This systematic review had a number of strengths. This review attempts to summarize the key programming and counseling features of exercise programs for cancer survivors that will likely increase adoption and maintenance. Although programming and counseling preferences represent one aspect of the design of PA programs, programs should be supplemented with social cognitive variables for long-term maintenance [38, 92]. Furthermore, this review highlights variations in PA preferences among cancer survivor groups and even within similar cancer types (i.e., breast cancer). Future research should consider examining preferences across the cancer care continuum to further tailor programs for cancer survivors before treatment, during treatment, and after treatment.

Generalizability may be limited given study response rates [47, 51, 61, 65, 66, 80]. Additionally, within studies that reported ethnicity, most participants were White, with only three studies assessing samples that were another ethnic majority. Most studies were conducted in the United States or Canada, with few studies conducted outside of North America [55, 60, 65, 70, 76, 78, 81–84]. Most of the literature reviewed has been conducted with mixed cancer survivor groups, and our ability to evaluate trends in preferences for cancer survivor groups that only had one study conducted with them was limited. Moreover, the format of the results in summarizing the most commonly preferred preferences may have concealed potential findings and erased certain nuances and PA preferences questions have not been tested for reliability and validity. A major weakness of current studies is that “PA counseling” is not well defined and it is unclear how individual studies are operationalizing this term. There are a multitude of potential applications and interpretations of the term such as relaying PA information, and prescribing PA. As a result, there is ambiguity in what is being assessed when evaluating PA counseling preferences.

Many cancer survivor groups are understudied and future research should be conducted with these populations. Research assessing PA and counseling preferences studies should focus on a sole cancer group instead of mixed cancer survivors, or separate the preferences of each cancer survivor group they are assessing, similar to the study conducted by Forbes et al. [69]. Because each cancer population may have unique barriers they face, to comprehensively create methods of increasing PA participation and adherence, researching PA preferences, along with barriers and facilitators to PA is necessary. Whitehead & Lavelle [83], Farrokhzadi et al. [65] and Rogers et al. [57] assessed barriers to PA participation while researching preferences, finding common barriers to include lack of time, priority or self-discipline, procrastination and feelings of tiredness or fatigue [57, 65, 83]. Evaluating barriers and facilitators among cancer survivors can assist researchers in designing optimal PA programs for this population. Future studies should also consider creating a PA preferences and counseling measure that can be used as a standardized preferences assessment and adapted across cancer survivor groups.

## Conclusion

Tailoring PA programs to cancer survivors’ preferences may be a potential step towards increasing participation and adherence. Considering the unique preferences of cancer survivors will create more engaging, accessible, and feasible PA programs for this population given that most cancer survivors are not meeting PA guidelines. The findings from this review suggest that many cancer survivors prefer moderate-intensity PA, walking, and morning-based programs. However, the wide variation in other PA preference variables suggests that it would be beneficial to establish multiple program options. Clinicians and researchers seeking to design interventions for cancer survivors should consider common preferences as a guide for the development and implementation of effective PA programs.

### Additional file

Additional file 1: **Table S1.** Appraisal Tool for Cross-Sectional Studies. **Table S2.** Consolidated Criteria for Reporting Qualitative Research. (XLS 29 kb)

## Appendix

**Table 3:** Search String Examples by Database

Database	Search String Example
PubMed	(“Exercise”[Mesh] OR exercis* OR “physical activity” OR “aerobic activity”) AND (“Neoplasms”[Mesh] OR neoplasm* OR cancer OR oncology OR “cancer survivor*” OR “cancer patient” OR remission) AND (preference* OR “counseling”[Mesh] OR “patient preference”[mesh] OR “patient preference” OR “patient satisfaction” OR “exercise preference*” OR counsel* OR “health behavior counseling” OR “distance counseling” OR “directive counseling” OR “online learning”)
SportDISCUS	(“Exercise”[Mesh] OR exercis* OR “physical activity” OR “aerobic activity”) AND (“Neoplasms”[Mesh] OR neoplasm* OR cancer OR oncology OR “cancer survivor*” OR “cancer patient” OR remission) AND (preference* OR “counseling”[Mesh] OR “patient preference”[mesh] OR “patient preference” OR “patient satisfaction” OR “exercise preference*” OR counsel* OR “health behavior counseling” OR “distance counseling” OR “directive counseling” OR “online learning”)
PyscINFO	((MJSUB.EXACT.EXPLODE(“Neoplasms”) OR MJSUB.EXACT.EXPLODE(“Oncology”) OR SU.EXACT(“Neoplasms”) OR “cancer patient” OR “cancer survivor” OR cancer OR neoplasms OR oncology)) AND((MJSUB.EXACT.EXPLODE(“Physical Activity”) OR MJSUB.EXACT.EXPLODE(“Exercise”) OR MJSUB.EXACT.EXPLODE(“Aerobic Exercise”) OR “aerobic activity” OR exercise)) AND (activity preferences* OR preferences* OR counsel* OR patient preference OR patient satisfaction OR exercise preference OR health behavior counseling OR distance counseling OR directive counseling OR online learning)
Web of Science	((Exercise OR exercis* OR “physical activity” OR “aerobic activity”) AND (“Neoplasms” OR neoplasm* OR cancer OR oncology OR “cancer survivor*” OR “cancer patient” OR remission) AND (preference* OR counseling OR “patient preference” OR “patient preference” OR “patient satisfaction” OR “exercise preference*” OR counsel* OR “health behavior counseling” OR “distance counseling” OR “directive counseling” OR “online learning”))
CINAHL	(“Exercise”[Mesh] OR exercis* OR “physical activity” OR “aerobic activity”) AND (“Neoplasms”[Mesh] OR neoplasm* OR cancer OR oncology OR “cancer survivor*” OR “cancer patient” OR remission) AND (preference* OR “counseling”[Mesh] OR “patient preference”[mesh] OR “patient preference” OR “patient satisfaction” OR “exercise preference*” OR counsel* OR “health behavior counseling” OR “distance counseling” OR “directive counseling” OR “online learning”)

**Table 4:** Literature Search Example - PubMed

PubMed Search	
**#**	Searches
1	“Exercise”[Mesh]
2	exercise or “physical activity” or “aerobic activity”
3	1 or 2
4	“Neoplasms”[Mesh]
5	neoplasms or cancer or oncology or “cancer survivor*” or “cancer patient*” or remission
6	4 or 5
7	preference* or counsel* or “patient preference” or “patient satisfaction” or “exercise preference” or “health behavior counseling” or “distance counseling” or “directive counseling” or “online learning”
8	3 and 6 and 7

## Abbreviations

AXIS tool: Appraisal Tool for Cross-Sectional Studies
BMI: Body Mass Index
COREQ: Consolidated Criteria for Reporting Qualitative Research
MeSH: Medical Subject Heading
PA: Physical Activity
PRISMA-P: Preferred Reporting Items for Systematic Reviews and Meta-Analyses Protocol
